# NIH Portfolio Allocation, Lemmings, and the Silent Spring: A Time-Capsule Commentary & Its Update

**DOI:** 10.12688/f1000research.1-5.v1

**Published:** 2012-07-20

**Authors:** Mark Boothby

**Affiliations:** 1 Department of Pathology, Microbiology & Immunology and Department of Medicine (Rheumatology), Vanderbilt University School of Medicine, Nashville, TN, 37232-2363, USA

## Abstract

With the release of the US President’s proposed budget for the Federal Fiscal year (FY) 2013, to start October 1, 2012, we’ve spun yet again into the mad vortex of an appropriation season. Fundamental re-thinks of how biological and medical research are prioritized and funded are urgently needed, but sadly appear to be unlikely unless the research and advocacy communities push harder and in a more unified manner. Early in the Obama presidency and the NIH Directorship of Dr Francis Collins, the FASEB Office of Public Affairs performed an analysis of trends in funding of R01 and other Research Project Grants and shared that with the Director and his office. Using the FASEB analysis, whose numbers drew on NIH data, an independent commentary (below) was submitted to (but not published in) Science. With the analysis a few years old, this older viewpoint is followed by updates that touch on how the trends have fared since early 2010 and comment on other aspects of the ongoing cull in biomedical research. In particular, data on some of the growth areas that continue to prosper at the expense of the ever-declining direct support for R01 science are discussed.

## Lemmings, and the Silent Spring [ *Feb. 2010*]

**Figure 1.**
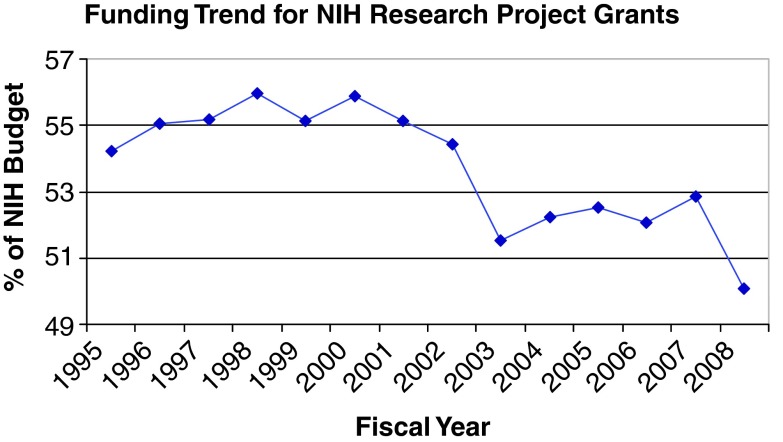
Trend in allocation of total NIH budget to NIH-designated Research-Project Grants (RPGs). Total NIH spending on RPGs (P01, P42, PN1, U01, U19, UC1, NIGMS P41, and all R-series) in each Federal Fiscal Year (FY) divided by total NIH budget. Analysis courtesy of FASEB, 2009, drawing on data from NIH Data Book.

**Figure 2.**
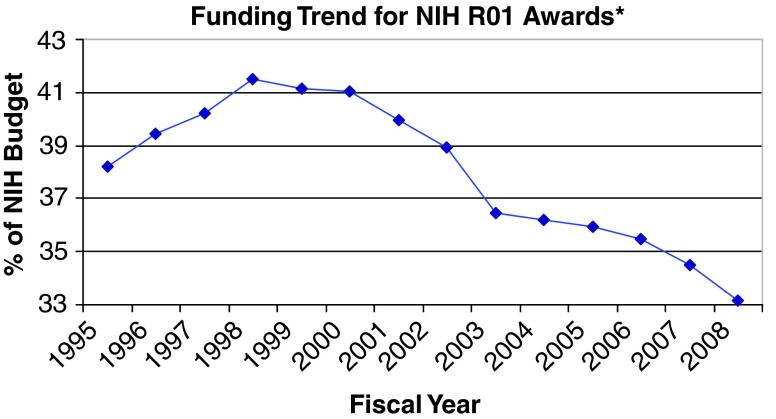
Trend in allocation of total NIH budget to R01/R01-equivalent grants. Total NIH spending on R01-equivalent grants (including the R29 program in earlier FY) in each Federal Fiscal Year (FY) divided by total NIH budget. Inset numbers highlight total numbers of R01 awards in ‘active’ status (a term that includes no-cost extensions in time) at the beginning of the post-doubling period and the last FY prior to ARRA. Analysis courtesy of FASEB (2009), using data from NIH Data Book.

Some milestones take on particular resonance and prompt reviews of trends – a New Decade; the first 200 days of a new National Institutes of Health (NIH) Director, and a Policy Forum from the Director ^1^. The long Congressional appropriation season is opening, and for the first time in recent memory the President has proposed a budget in which the NIH might lose less ground to inflation. Accordingly, it is timely to open a discourse regarding changes in management of the NIH enterprise over the past 8–10 years. The data ( Figure 1 and Figure 2) should catalyze an analysis of the premises and likely impact of these changes on health-related research, education and progress in the USA. The multitude of productive research teams and themes extinguished in recent years suggests the view that trends in biomedical research funding allocations are changes analogous to those heralded in "Silent Spring" ^2^. This little-discussed toll should be a part of analyzing how the US is faring in its need to stay the locomotive of discovery and progress in health.

The force and persistence of the underlying trends emerged in data that capture overviews of directions the NIH has taken. They include the last federal fiscal years (FY) that can reliably picture management trends of the NIH prior to the short-term impact of the American Reinvestment and Recovery Act (ARRA). A few data snapshots bear particular comment. Damage continues to worsen as the NIH appropriation loses ground to inflation year after year in the "post-doubling era" ^3^. However, it is equally true that the NIH decreased its allocation of funds to the gold standard of investigator-initiated research (the unsolicited R01 grant) by $300,000,000 for FY2008 despite an overall budget $1.5 billion higher than in FY2004. As such, a rising tide seems to have been scuttling the boat rather than lifting it. The full post-doubling era (FY2003–2008) does not offer a much more encouraging picture – the NIH budget increased by $2.8 billion, and only 3% of that increase was allocated to the R01 program.

These and other changes at NIH appear to embody a philosophy during the past 8–10 years that, in the present era, the foundation of successfully advancing human health has somehow changed and the rules are different. This view culturally resembles thoughts about business and finance during the same era: ‘now things are different’, i.e., advances and the frontier of new investment tools meant that the fundamental rules of finance and risk had been profoundly changed. Analogously, the evolving NIH philosophy appears now to tilt more toward larger programs, central management, and central direction as a paradigm based on the premise that ‘the rules are different’ now compared to earlier days, and that ‘innovation’ or ‘translation’ didn’t occur under investigator-initiated mechanisms or ‘the old way’.

The data represent a significant turn away from a cornerstone of the NIH during its long period of success. The first question asked by working scientists after viewing these data (striking declines in RPGs as a whole, and even more strikingly in funding of R01s, the bedrock of NIH success) is "So where has all that money been going?" Different scientists seem to have best guesses (most commonly, large contracts and centers managed centrally by the NIH), but a striking thing is that these are guesses: the answer is not readily apparent despite a statutory mandate for greater transparency. To provide a rationale for such a large shift in portfolio allocation, a key question in management planning of each independent Institute must be: "What is the evidence of disproportionate success from this shift of funding focus?"

Historically, the primacy of the Research Project Grant and the unsolicited R01 were central to a research culture that promoted innovation and creativity from the widest and most diverse range of scientists possible. This tradition was, and remains, absolutely vital. Great medical advances stem from scientists who originally were not at the top of the food chain working on questions that would originally not have been considered among the ‘hot areas’ before years of groundwork followed by a breakthrough discovery. Peer review on its surface may seem to engender conservatism because study sections take their role as guardians of precious taxpayer dollars seriously, but R01 grants embody key features essential for innovative discoveries and long-term success in approaching the unknown. Vital qualities of the R01 grant that drive this sort of progress are (i) the awards are renewable, multi-year grants of sufficient duration and (ii) flexibility in allowing adjustments of direction. Especially when unsolicited, the duration and flexibility encourage innovation and harness the creativity of tens of thousands of investigators with ideas that current paradigms or central planners would not likely imagine, let alone prioritize. In short, R01 science promotes innovation.

At the same time, a sufficiently large and diverse portfolio is vital for "consolidation", i.e., the validation and incremental extension or solidification of apparent discoveries. This part of science is as integral to progress as innovation but gets short shrift by implication. To paraphrase Thomas Edison, one of the most successful inventors and innovators in US history, progress is "1% innovation and 99% perspiration". A balance is needed between innovation (or ‘transformation’) and ‘consolidation’ in advancing human health by ‘reducing innovation to successful practice’ by validation and extension (i.e., consolidation). Will a NIH moving to centrally managed large contracts and large infrastructure projects – with innovation in a fringe minority of its funding and an ever-dwindling amount of consolidation – be the optimal model for advancing health? Similarly, shifting funds to translation raises the urgent question: who but the NIH will maintain the US’ lead in basic investigation, and how deep will the understanding of mechanisms underpinning translated ideas be? The result of the previous cultural period is a proven success. Whether the changes at the NIH might improve on that record is a central issue for skepticism and open discussion.

Despite much-discussed and glaring shortcomings in the delivery and quality of medical treatment in the US, we have witnessed an extraordinary fall in death rates from ischemic and atherosclerotic vascular diseases and, for all the concern that progress is not even greater, major advances in the fight against cancers. In the context of a paradigm shift for the NIH, there is a need for broad-based input addressing hard questions. Foremost among these is whether diverting money from unsolicited research project grants and R01s into large-scale programs that ostensibly will ‘de-risk’ agents for pharma or hew to 5 year plans for identification of new therapeutics is realistic or viable. The continuing force of Vannevar Bush’s germinal report rings as true now as through the past 65 years:

*"The striking advances in medicine [. . .] have been possible only because we had a large backlog of scientific data accumulated through basic research in many scientific fields", "Scientific progress on a broad front results from the free play of free intellects, working on subjects of their own choice, in the manner dictated by their curiosity for exploration of the unknown. Freedom of inquiry must be preserved under any plan for Government support of science", and "Basic research is a long-term process – it ceases to be basic if immediate results are expected on short-term support. Methods should therefore be found which will permit the agency to make commitments of funds from current appropriations for programs of five years duration or longer."*

With this latter point in mind, one striking trend within the R (investigator-initiated) series of NIH grants is the trend towards ever-larger numbers of R21 (2 yr) awards as the number of R01-equivalent grants falls.

The mission of the NIH and its great opportunities and promise were beautifully summarized by the NIH Director but two items were notable in the Director’s Research Agenda ^1^. First, while observing that creative insights of individual investigators are the foundation of success in advancing human health, it states that ‘increasingly investigators are working in teams’ and ‘a careful balance is needed between individual investigator-initiated projects and large-scale community resource-generating efforts’. In fact, investigators in an ‘R01- (& P01-) centric’ era of NIH culture involved teams of investigators and the NIH allocation of funds always involved a careful and judicious balance of large projects, large multi-center clinical science, and other team or infrastructure investigations. The questions for NIH and its Institutes are: what is the appropriate balance; why has the balance been changed at present; and, since a shift went on for 8–10 years, what evidence is there in terms of rigorous, independent ‘cost-benefit’ analyses to support continuing the change in allocation?

The most urgent priority, and most vitally needed remedy for these potentially devastating effects on the biomedical research, education and training enterprise, is for Congress to sustain and expand increases in funding of the NIH. However, appropriations in the post-doubling era have been framed in a broader fiscal picture. The Federal fiscal picture for years to come will look worse than it did during 2000–2009. The NIH appropriation for this fiscal year and the President’s proposed budget – with a 3.2% increase and a pie chart suggesting 53.5% of the appropriation may go to RPGs – might be a cause for some optimism unless the rising tide continues to scuttle the RPG/R01 boat. For instance, the proposed impact from a $1,000,000,000 increase would be a drop in the pool of money for new/competing renewal RPGs (200 less new grants for what is termed a high priority). A comparison of the numbers for RPGs to the period 2004–2008 is bleaker, and data trends tracking the fraction of RPG money going to R01-equivalent awards suggests an even more dire prognosis. NIH and the health research community need a better plan for how to deal with increases less than what is genuinely needed, but more than the cuts that so many government agencies and individual citizens face. A central issue for such planning is to discuss the question whether the disproportionate cuts in the RPG and R01 programs are wise choices. Alternatively, is this the time for tough choices be made to move back toward the portfolio allocations that prevailed as NIH’s norm in its period of successes proven to advance human health?

At present, large swaths of the research community are in a state akin to an infection at the stage when it is controlled but approaching the point of tipping into systemic shock and irreversible organ damage. In weighing costs and benefits of the large change in portfolio allocation at NIH (i.e., progressively underweighting the R01 component), the long-term effects on US health are little discussed. Part of what drove the very best in American medical training was the involvement of a large set of active researchers in medical training at all levels. Set-backs to training medical professionals in the advanced science of health will be set-backs to progress in health itself. The damage to that culture needs to be reckoned among the secondary consequences of the NIH shifts even if the statutory mission of NIH does not include direct support of medical or health education other than the NRSA program. As primary damage, what looms is a ‘cull’ eliminating further thousands of trained team leaders (faculty active in research and education) and unique biomedical research avenues with important contributions hanging in the balance. Perhaps witnessing this cull of established research teams will inspire talented and more innovative minds to flock to biomedical research careers?

Sadly, in light of these trends the impression many active, talented, productive mid-career biomedical researchers have formed as to how their activities are viewed by the NIH is that lemmings offer the best metaphor. This species is subject to explosive population growth and sudden, devastating declines. While enshrined in popular imagination as committing mass suicide by jumping off a precipice, in fact the lemmings were actively pushed off the edge to make a film. NIH funding will always be limited relative to the opportunities, but great opportunities are best realized by an optimal portfolio allocation. For the long-term health of the country, let’s hope that cohorts of scientists won’t be herded off "The Cliff."

## Two years later – an update [ *Feb. 2012*]

**Figure 3.**
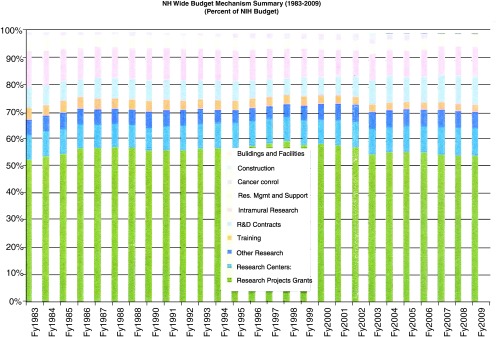
Trend in allocation of total NIH budget to NIH-designated Research-Project Grants (RPGs) – OER analysis of 2010. Total NIH spending on RPGs (P01, P42, PN1, U01, U19, UC1, NIGMS P41, and all R-series; green bars) as well as other components of the research funding enterprise, for each Federal Fiscal Year (FY) 1983 to 2009, divided by total NIH budget. Analysis courtesy of NIH OER (abstracted from a July, 2010 letter replying to input).

**Figure 4.**
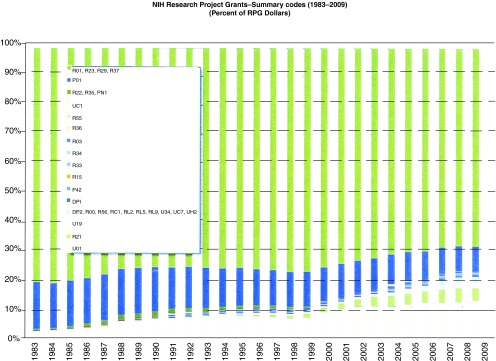
Trend in allocation of total NIH budget to NIH-designated Research-Project Grants (RPGs) – OER analysis of 2010. Total NIH spending on various components of the RPG pool (P01, P42, PN1, U01, U19, UC1, NIGMS P41, and all R-series) for each Federal Fiscal Year (FY) 1983 to 2009, expressed as a % of the overall (but shrinking) RPG allocation. R01, green bars. Analysis courtesy of NIH OER (abstracted from a July, 2010 letter replying to input).

A first question to consider in relation to the original analysis is about the data accuracy. In mid-2010, the NIH Office of Extramural Research (OER) kindly provided its analysis of the numbers ( Figure 3 and Figure 4, scans of 2010 OER data labeled as Figure 2 and Figure 4). While apparently accounting for some monies (SBIR/STTR) a bit differently, the basic trends were similar to the FASEB analysis (for instance, a drop in R01-funded research from around 42.5–43% of total NIH budget in the 15 year period FY1988–2002, to 38% in FY 2009). What dynamic drives the sort of change in portfolio allocation identified in these data? One thoughtful angle on why so many of the Institutes and Centers of the NIH trend in this way was spelled out in late 2011 in a marvelous opinion piece in Genome Biology ^4^.

In the spirit of Congressional report language in NIH appropriations, the OER had several enlightening updates as part of soliciting blog-style input. Congress correctly indicated that constantly whittling down award sizes and steadily decreasing the numbers of grant awards does not provide a recipe for success. Moreover, the troll lurking under the bridge for FY2013 is that, with the predictable failure of the Gang of Twelve ("Joint Select Committee on Deficit Reduction"), the NIH faces the possibility of a sequestration that would cut its budget by over 7.5%. NIH OER offered some insights into ways to model how much various changes could help, framed in an online solicitation, " *How Do You Think We Should Manage Science in Fiscally Challenging Times*?" One of the most striking points of data was that the probability of success on applications increased as application sizes get larger.

More recently, OER provided further data that support the Lemming Theory of Biomedical Research(er) Management. This thoughtful piece is noteworthy in documenting that just for one year, FY2010 to FY2011, the number of new/competing Research Project Grant awards dropped from 9432 (not including ARRA awards, which have now gone away) to 8776. That fall represents a decline of about 7% in just one year, and of course the end of an ARRA means even more projects consigned to the ashbin! Since NIH reports that, within the extramurally funded community, about 80% of funded PIs lead only one RPG, it is likely that 676 ‘lost RPGs’ represent a sizable cull in the PI community.

These points bring one back to the President’s proposed FY2013 budget. The budget as a whole has virtually no chance of being adopted by Congress – especially as tough re-election campaigns are in gear for the President and Congress, with fiscal and economic issues as major fronts in the war ahead. Nonetheless, the NIH is an agency managed by the Executive Branch so the budget proposal reflects a mindset that will continue to prevail – barring sufficient pressure to drive change.

A brief pause is in order to think happy thoughts. If one forgets about the possibility of sequestration, the budget at least requests no decrease. Many other agencies will be cut. That said, the executive summary tips a glance at the cards in noting that the pool of money for RPGs should go down (albeit a mere $26,000,000 – aka ~50 10-module R01 grants) while continuing cost escalation in R&D contracts (up $108,000,000) and the NIH intramural research program (up $22,000,000). That the request continues the trend noted in the 2009 FASEB analysis requires looking at the numbers. Strikingly, money for the RPG is down to 51% of NIH total funding (closer to 54% if one includes SBIR/STTR). Even with the shaky assumption that the R01 will hold its ground at 69% of RPGs (FY2009, down from ~75+% in the glory days of the NIH), this budget would drop the R01 below 37% of the overall NIH budget (down from ~43% in the FY1988–2002 period). Fear not, however! Despite the drop in requested monies, "The number of new or competing RPGs would increase by 672, resulting in an estimate[d] success rate of 19 percent". Truly, we live in magical times!*

Seriously folks, the bottom line on how the administration proposes to achieve this increase is to cut awards in their "off-years" (non-competing continuation awards), laudably to cut out the preferential treatment of non-modular awards (i.e., eliminate the cost-escalations that they, but not non-modular awards, receive), and push the mix toward smaller and shorter Research Project Grant awards. Incidentally, a good by-product of the OER piece is that it gives some clues on ways to use the NIH RePORTER tool to get at some of the data on one’s own. For the key insights into trends after breaking total costs down into direct vs. indirect cost components, and extramural vs. intramural funding, the NIH budget text is the place to go for further gems. For instance, taking the extramural funding pie as a whole, the data show that the aggregate trend in Facilities & Administration (F&A, or "indirect") costs continues a long-suspected climb. Sure, these costs have "only" increased their share from ~37.2% (FY2002 & 3) to 39% (FY2011). And, sure, the intramural program has "only" climbed 0.6% in its share of the overall NIH budget (10.4 to 11%, which on a $30 billion base means $180,000,000). But 1% of a $22 billion extramural research effort is $220,000,000, or ≥ 500 more 10-module R01 grants. Add to that the money escalating R&D contracts but not basic science research, and one arrives at cost escalations of as much as $725,000,000 in a post-doubling era of "flat funding", which have to come at the expense of direct funding for R01 science.
