# Supplementary material for: NIH Portfolio Allocation, Lemmings, and the Silent Spring: A Time-Capsule Commentary & Its Update
Source: F1000Res. 2012 Jul 20;1:5. [Version 1] doi: 10.12688/f1000research.1-5.v1 (PMC3752635; doi:10.12688/f1000research.1-5.v1)
Supplement: Peer review history

# Peer review: Referee response for version 1

**Published:** 2012-08-05

**Authors:** Mark Bix

**Affiliations:** Department of Immunology, St. Jude Children's Research Hospital, Memphis, TN, USA

Mark Boothby’s article on the deplorable state of current NIH investigator-inititated research funding is right on target.

I add my voice to the chorus of scientists witnessing and lamenting the growing struggle to maintain research funding. As this state of funding crisis continues, I believe that young talent will increasingly flee from the biomedical research enterprise. Eventually, the United States will cede to other nations the ability to generate the next bumper crop of biomedical discoveries.

I have read this submission. I believe that I have an appropriate level of expertise to confirm that it is of an acceptable scientific standard.

# Peer review: Referee response for version 1

**Published:** 2012-08-02

**Authors:** Alan Schechter

**Affiliations:** Molecular Medicine Branch, National Institute of Diabetes and Digestive and Kidney Diseases (NIDDK), Bethesda, MD, USA

This commentary on NIH budgeting is a cri de coeur from a well-respected MD/PhD immunology investigator about his perception of the untold damages that recent changes in NIH budgeting will likely have on “the long-term health of the country”.

His views, which are likely representative of a great many NIH-supported scientists, appear to focus primarily on the cyclic changes in dollars of support leading to continued “cull[ing] of established research teams” (i.e., the lemmings) and that these trends are (for reasons less clear to me) “analogous to those heralded in Silent Spring”. Although one sympathizes strongly with the concerns of the author, the manuscript is, in my opinion, not a serious contribution to the long overdue but badly needed policy discussion of how NIH and other medical research funding should be structured. Cynically, one might even say that the paper is yet another example of the “sky is falling in” response to any suggestion that the author’s source of support may be undergoing changes. The author surprisingly points out in the abstract that his paper was rejected by Science two-and-a-half years ago but resubmits it now with some updating; I think it still is not a substantial contribution to these policy issues.

The figures and data in the paper show NIH numbers on different types of funding mechanisms and grants over the last several decades and, although the author does not explain which of these he approves of, the implication seems to be that there has been some relative decline in “the gold standard RO-1″ as calculated in various comparisons. This change apparently is apparently the cause of our current crisis. The author seems unaware that these changes, which in my analysis are really quite small, have occurred in the last decade-some initiated by the NIH leadership and some from other factors, such as congressional mandates to study child development- because of increasing skepticism about the results of the overall program. Thoughtful people and organizations have questioned whether the policies of the “golden years” are still as fruitful-whether measured by changes in public health parameters, new pharmacological agents or more subjective evaluations of our successes in dealing with the major chronic illnesses. In this era of genomics, proteomics and other aspects of “big biomedical science” the author is silent on how these needs are to be balanced with his desire for “a hundred thousand flowers”.

The strange history of this paper also results in its being largely outdated at this point. Specifically, the author does not discuss the very recent creation of the National Center for the Advancement of Translational Sciences, the slightly older Centers for Translational Science Awards or the components of the ten year old NIH Roadmap (now the Common Fund). Although perhaps not in the author’s ken, these new programs have been created in response to needs articulated by other investigators. Even FASEB, which presumably supported the earlier draft of this paper, has just issued a “white paper” in support of the new NIH initiatives in translational research. I wonder if this surprises the author.

I would recommend the author uses the rigor of his approach to other research problems to make his contribution to what will be a very important continued debate in the several decades ahead, especially if political and economic trends continue to limit greatly funds for medical research, about the relationship of basic and applied science, little vs big science, what planning is needed and is valuable, and many other questions.

I have read this submission. I believe that I have an appropriate level of expertise to confirm that it is of an acceptable scientific standard, however I have significant reservations, as outlined above.

# Peer review: Referee response for version 1

**Published:** 2012-07-31

**Authors:** Nina Papavasiliou

**Affiliations:** Laboratory of Lymphocyte Biology, The Rockefeller University, New York, NY, USA

This well documented commentary goes hand in hand with the very recently published Science article (much shorter by necessity). Both pieces are raising a RED alert on the significant and PRESENT danger of the whole NIH system, which has been significantly degraded during the last decade, now literally going off the cliff.

Clearly, a major reorganization is in order, and this will happen by necessity, with or without the input of working scientists. To make an analogy to current affairs, the federally funded scientific enterprise in the USA is functionally bankrupt, and the only choice remaining is between an orderly reorganization, or, a disorderly collapse. Let’s hope for the former.

I have read this submission. I believe that I have an appropriate level of expertise to confirm that it is of an acceptable scientific standard.

# Peer review: Referee response for version 1

**Published:** 2012-07-31

**Authors:** Sebastian Fugmann

**Affiliations:** Laboratory of Cellular and Molecular Biology, National Institute of Aging, Baltimore, MD, USA

This Commentary by Mark Boothby adds to the increasingly louder voices expressing their concerns about the trajectory of NIH-funded biomedical research. It takes the plain numbers and places them in the context of the day-to-day reality in a R01-based research group which, as the title suggests, might be a species going extinct.

While some critics might portrait this manuscript as a complete exaggeration and an unwarranted “the end of the world is near” song, the underlying facts and numbers are quite clear – the number of old-school R01-supported labs is slowly but surely shrinking. Allocation of public funds for scientific research is a political decision, and as such they are a compromise between opposing force that try to achieve their very own agenda – on the one side the supporters of large goal-oriented research programs (largely the powerful leaders of such huge and expensive projects – while many scientist would love to be such leader, very few would simply dream of being only a tiny little almost dispensable piece of such big scientific machinery) on the other hand the traditional principal investigator who follows his own intuition and fights every five years to get his R01 renewed to continue his work. Thus lobbying to reconsider the ongoing changes is a legitimate action, and this commentary spells out what has been a major topic of conversations during coffee breaks at scientific conferences and grant study sections.One question that remains, however, is whether the arguments of scientists have a stronger impact when presented in a somewhat dull and sober style like a traditional research paper, or as it is done here in a more accessible fashion, including a healthy dose of cynicism. Clearly, this is up to the reader to decide, but even if one does not like the tone, hopefully some of the message will stick.

I have read this submission. I believe that I have an appropriate level of expertise to confirm that it is of an acceptable scientific standard.
